# Moderate plant water stress improves larval development, and impacts immunity and gut microbiota of a specialist herbivore

**DOI:** 10.1371/journal.pone.0204292

**Published:** 2019-02-20

**Authors:** Elena Rosa, Guillaume Minard, Johanna Lindholm, Marjo Saastamoinen

**Affiliations:** 1 Organismal and Evolutionary Biology Research Programme, University of Helsinki, Helsinki, Finland; 2 Dept. of Ecology and Genetics, Uppsala University, Uppsala, Sweden; 3 University of Lyon, Lyon, France, University Claude Bernard Lyon 1, CNRS UMR 5557, Laboratory of Microbial Ecology, INRA UMR1418, Villeurbanne, France; Institute of Plant Physiology and Ecology Shanghai Institutes for Biological Sciences, CHINA

## Abstract

While host plant drought is generally viewed as a negative phenomenon, its impact on insect herbivores can vary largely depending on the species involved and on the intensity of the drought. Extreme drought killing host plants can clearly reduce herbivore fitness, but the impact of moderate host plant water stress on insect herbivores can vary, and may even be beneficial. The populations of the Finnish Glanville fritillary butterfly (*Melitaea cinxia*) have faced reduced precipitation in recent years, with impacts even on population dynamics. Whether the negative effects of low precipitation are solely due to extreme desiccation killing the host plant or whether moderate drought reduces plant quality for the larvae remains unknown. We assessed the performance of larvae fed on moderately water-stressed *Plantago lanceolata* in terms of growth, survival, and immune response, and additionally were interested to assess whether the gut microbial composition of the larvae changed due to modification of the host plant. We found that larvae fed on water-stressed plants had increased growth, with no impact on survival, up-regulated the expression of one candidate immune gene (pelle), and had a more heterogeneous bacterial community and a shifted fungal community in the gut. Most of the measured traits showed considerable variation due to family structure. Our data suggest that in temperate regions moderate host plant water stress can positively shape resource acquisition of this specialized insect herbivore, potentially by increasing nutrient accessibility or concentration. Potentially, the better larval performance may be mediated by a shift of the microbiota on water-stressed plants, calling for further research especially on the understudied gut fungal community.

## Introduction

Despite the general expectation of plant water stress negatively impacting herbivores relying on the plants, the responses of insect herbivores to plant drought vary. The variation in response depends on the duration and frequency of the stress, as well as on the insect feeding guild or species [1]. However, continuous water stress is generally expected to have a negative impact [1]. Indeed, the degree of the water stress is also important, and extreme desiccation killing the host plant is likely to have a negative impact on herbivore fitness. Some insects clearly benefit from drought events, as is evident by insect outbreaks taking place simultaneously with plants undergoing water stress [2,3]. However, these responses of insect outbreaks are not necessarily linear [3]. Similarly, the performance of insects from various feeding guilds has been shown to not increase linearly with plant watering condition, with worse performance on intermediately water-stressed plants compared to either well-watered and heavily stressed ones [4]. In addition, herbivore preference and performance do not always match, as shown in the specialist *Pieris brassicae* butterflies preferring well-watered host plants, but performing better on water-stressed ones [5].

One key indicator of insect condition, often overlooked in insect performance studies, is immunity. Insect immunity can vary in relation to diet abundance and quality [6,7], the presence of plant defense compounds [8–10], and microbial composition of the plant (reviewed in [11]). Moreover, energetic costs due to the activation of insect immunity are expected to rise on suboptimal diets [12,13]. Furthermore, while considering insects and their host plants, we should bear in mind that they do not perform individually, but rather as holobionts composed of the insect or plant host and its microbial community [14]. Indeed, microbial communities play a role in insect digestion, immunity, and development, amongst others [15–20]. Microbiota composition varies with host habitat and food source [21]. Contrarily to other insects, the impact and spread of extracellular symbionts is quite limited in Lepidoptera [22], with recent work suggesting that bacterial communities are often acquired through food and environment [22,23]. Therefore, changes in the plant microbiota due to climatic factors might lead to drastic changes in the microbiota of larvae. Despite a large body of literature on the effects of plant condition on insect development, the impact on other indicators of insect condition, as the ability to maintain an adequate immune response, and a balanced gut microbial composition remain largely unexplored. With this study, we aim at opening a discussion on the potential interactions among these aspects of insect condition less commonly tested.

We used the Finnish Glanville fritillary butterfly as a model system to investigate the effects of water-stressed host plants on the performance of larvae feeding on them. Based on estimates from the field and long-term data [24,25], reduced precipitation is expected to substantially impact population dynamics. Pre-diapause larvae are very sessile and have limited ability to move among host plants. Hence, their survival strongly depends on the condition of the host plant they develop on. Whether the suggested poorer performance of larvae during drought events is the result of starvation due to extreme plant desiccation, or also of subtler changes in host plant quality driven by moderate water stress remains unknown. We reared a set of pre-diapause larvae on host plants subject to moderate continuous water stress or constantly well-watered and compared their performance. We assessed larval growth rate and survival but also the impact of changes in plant quality on less commonly tested potential indicators of larval condition: immunity and the composition of the gut microbial community (bacteria and fungi).

## Material and methods

### Experimental design

The seeds of the host plant, *Plantago lanceolata*, were collected in the Åland Islands in 2015 (Seglinge, 60°11’45.6”N 20°42’14.4”E; the collection site is publicly owned and no permits are required to obtain the seeds). The plants were reared in a greenhouse with artificial lighting (16/8h L/D). We introduced moderate water stress on the host plants as follows: a batch of 74 plants was daily watered with 20 ml for the “water stress” treatment, whereas a batch of 38 plants was watered with 40 ml for the “well-watered” treatment. The treatments resulted in clearly distinctive plant phenotypes, with water-stressed plants producing lower biomass and wilted leaves, and well-watered plants having high biomass and vigorous leaves (personal observation). Note, however, that this was not formally tested in the present experiment. We used *M*. *cinxia* larvae of eleven separate families, which were the F1 of individuals collected in Åland in 2015. Forty 24h-old larvae per family were assigned to each treatment (water stress vs. well-watered), and reared in sterile petri dishes. Larvae were fed daily a 2.25 cm^2^ leaf piece corresponding to their given treatment until the 4^th^ instar, while 4.5 cm^2^ leaf pieces were used after the 4^th^ instar. To minimize the effects of leaf tissue deteriorating once cut from the plant and placed in the petri dish with the larvae for 24 h, 0.35 ± 0.05 ml of water was added on the surface of the leaf pieces. Providing moisture to the rearing environment is a standard daily procedure with *M*. *cinxia* larvae as it increases humidity and thus prevents both leaf tissues and larvae from drying out. Developmental time was estimated as the date when ½ of the larvae within a petri dish molted to a new instar. The larval growth rate was determined by dividing the average body mass of larvae in each petri dish by the development time until the 5^th^ instar. Survival was assessed once the larvae reached the 5^th^ and diapausing instar. At least 25 larvae per treatment per family survived until the 5^th^ and diapausing instar, except for one family. At this stage, all the larvae were weighed, and five randomly chosen ones were snap-frozen and stored at -80°C for later immune gene expression analysis. Because assays testing the immune response like phenoloxidase activity (see below) can be better interpreted in the light of an actual infection challenging the immune system, we infected half of the individuals with an entomopathogenic bacterial strain as a positive control. The remaining half was still punctured but without bacteria. We punctured with a microneedle the penultimate proleg of 20 diapausing larvae after 24h of starvation: ten were challenged with *Micrococcus luteus* (250 mg/ml lyophilized cells), and ten with 1X PBS (Phosphate-buffer saline solution, Gibco-USA) as a control. Twenty-four hours later these larvae were snap-frozen and stored at -80°C for further analysis.

### Phenoloxidase activity

The phenoloxidase enzyme is an indicator of insect immunocompetence [26]. As gut dissection caused complete bleeding, we could not restrict the assay to the haemocoelic PO activation alone, but we assayed whole body homogenate of ten pooled larval carcasses. The measurements were based on modified methods by [26]. Pools of ten larval carcasses per family per treatment were weighed and placed in a screw-cap tube with 20 μl of ice-cold PBS per mg measured and crushed with a metal bead in a Bead-ruptor machine (30 rps for 1.5 min). The tubes were centrifuged at 13 000 rpm for 10 min at 4°C, and 20 μl of the supernatant was transferred in a new Eppendorf tube with 20 μl of ice-cold PBS. The assay was performed in 96-well plates and the increase in absorbance due to melanin production was measured every minute for 100 min at 490 nm and 30°C with an EnSpire microplate reader (PerkinElmer). Each well included 70 μl of ice-cold distilled water, 10 μl of ice-cold PBS, 7.5 μl of a sample, and 10 μl of 10 mM L-Dopa solution. The PO activity was assessed as the slope of the reaction curve during the linear phase (V_max_).

### Immune gene expression

Five individuals per family per plant watering condition were assessed for immune gene expression. Typically, immune responses are tested with an immune challenge serving as positive control. Due to data limitation, this was not possible; hence immune gene expression is assessed only in response to plant water stress, hence providing a measure of the basal immune level. However, previous work shows upregulation of most of the tested genes under bacterial challenge [27]. To restrict the immune gene expression to body tissues comparable to those used to assay the PO activity, whose gut was dissected and not included in the immune assay, the qPCR was also performed on individual carcasses dissected as described above. RNA was extracted by homogenization of the samples in 1 ml of Trisure (Bioline-Germany). The qPCR targeted seven immune genes following methods by [27]: Lysozyme C, prophenoloxidase (proPO), attacin, peptidoglycan recognition protein LC (PGRP-LC), β‐1,3‐glucan recognition protein (βGRP), serpin 3a, and pelle, as well as two housekeeping genes: mitochondrial ribosomal protein L37 and S24. Phase separation was performed by supplementing the mixture with 200 μl of Chloroform and centrifuging the mixture at 12 000 g for 15 min at 4°C. The aqueous phase was then collected, precipitated in 0.5 ml of isopropyl alcohol for 10 min at 12 000 g and 4°C for 10 min. The RNA pellet was washed with 1 ml of 75% ethanol and centrifuged for 5 min at 4°C. RNA pellets were dried and dissolved in 20 μl of DEPC-treated water (Ambion, USA). Dissolved RNAs were then treated with DNAse I (Thermo Fisher Scientific, USA) and reverse transcribed into cDNA using iScript cDNA Synthesis Kit (Bio-rad, USA) and the manufacturer’s conditions. The qPCR was performed with the following program: 95°C for 3 min × 1 cycle, 95°C for 12 s × 40 cycles, 59°C for 1 m × 40 cycles, and melt curve generated from 65°C to 95°C with an increment of 0.5°C for 5 s. The RT-qPCR values were transformed into a relative expression according to the 2^-ΔΔCt^ method using the housekeeping genes Ct values and the immunity genes Ct values of individuals fed on well-watered plants as the baseline.

### Microbial community structure

All larvae challenged with *M*. *luteus* and PBS were individually washed three times with 500 μl of 1X PBS (Ambion- USA), surface-sterilized in 70% ethanol and washed five times in 1X PBS. Midguts were dissected under sterile conditions in 1X PBS. Ten pooled midguts were used per condition and per family. DNA was extracted from pooled midguts using Qiagen DNeasy Blood and Tissue kit (Qiagen-Germany) with a previously adapted protocol [28]. Bacterial and fungal Automated Ribosomal Intergenic Spacer Analysis (ARISA) were performed with primers amplifying the intergenic regions of 16S-23S rDNA (bacteria) or 18S-28S rDNA (fungi). Bacterial ARISA (b-ARISA) was carried out in triplicates for each sample with the primers ITSF (5’FAM -GTC GTA ACA AGG TAG CCG TA-3’), ITSReub (5’-GCC AAG GCA TCC ACC-3’) and the fungal ARISA was conducted with the primers 2234C (5’HEX-GTT CCG TAG GTG AAC CTG C-3’) and 3126T (5’-ATA TGC TTA AGT TCA GCG GGT-3’) [29,30]. PCR was conducted with a reaction mixture containing 1X of Q5 buffer (New England Biolabs, USA), 1X of High-GC enhancer (New England Biolabs, USA), 200 μM of dNTP (Thermo Fisher Scientific, USA), 500 nM of each primer and a total of 0.12 mg×ml^-1^ of Bovine Serum Albumin (New England Biolabs, USA), 0.06 mg×ml^-1^ of T4gene32 (New England Biolabs, USA) in a final volume of 25 μl. Amplifications were conducted on an ABI Veriti thermal cycler (Applied Biosystem, USA) with 3 min of denaturation at 94°C, 30 cycles of 45 s at 95°C, 1 min at 55°C, 1 min 20 s at 72°C followed by a final elongation of 1 min 20 s at 72°C. Replicates of each sample were first controlled on a 1% agarose gel for positive amplification, then purified with a PCR Clean-up kit (Macherey-Nagel, Germany) following the manufacturers’ recommendations, quantified with NanoDrop (Thermo Fisher Scientific, USA) and diluted at a concentration of 10 ng×μl^-1^. Capillary migrations were performed on a 3730XL Bioanalyzer (Applied Biosystem, USA), using 4 μl of each sample, 10.8 μl of Hi-Di formamide and 0.2 μl of GS 1,200 LIZ size marker (Thermo Fisher Scientific, USA). The fluorograms were analyzed with the software Genemapper 4.0. The signals comprised between 100–1000 bp were selected, binned to windows of 5 bp and transformed into relative fluorescence intensities (RFI) using a previously published method [31].

### Statistical analysis

The effect of the plant treatment on growth, survival, phenoloxidase activity and immune gene expression was estimated with a linear mixed model approach with treatment as an explanatory variable and family as a random variable. We tested the impact of the random effect ‘family’ by calculating intraclass correlation coefficients (ICC) of all the models as a proxy of the proportion of phenotypic variation explained by the larval genetic background. All models were run with packages *ade4* and *LmerTest* in R [32–34].

The b-ARISA and f-ARISA normalized matrix were used as an input to compare the bacterial and fungal communities among the midguts of *M*. *cinxia*. The diversity analysis was conducted with the package *vegan* in R [32,35]. The Shannon index (H’) was used to compare the within-individual bacterial or fungal diversity (also called α-diversity). This index is defined as $H^{\prime }=-\sum_{i=1}^{S}p_{i}.{log}_{2}p_{i}$ where *i* is an individual taxon, *S* is the taxa richness and *p*_*i*_ is the relative abundance of a given taxon compared to the total abundance of all the taxa. Shannon indices were compared among individuals and conditions using linear mixed models with the family used as a random effect. The among-sample difference in bacterial or fungal communities (also called β-diversity) was measured through the Bray-Curtis dissimilarity distance (BC). This distance is defined as ${BC}_{ij}=\frac{\sum_{k=1}^{S}\left|p_{ik}-p_{jk}\right|}{\sum_{k=1}^{S}{(p}_{ik}+p_{jk})}$ where *S* is the species richness, *p*_*ik*_ is the relative abundance for the taxon *k* in the sample *i* and *p*_*jk*_ is the relative abundance for the taxon *k* in the sample *j*. The Bray-Curtis distances among the samples were represented using a non-metric multidimensional scaling (NMDS). The potential impact of the water stress plant treatment and pathogen infection on the Bray-Curtis distances were tested with a permutational analysis of variance (*adonis*-ANOVA) and plotted with a Canonical Analysis of Principal coordinates (CAP). These methods are described with more details in the manual of the *vegan* package [35].

## Results

**Fig 1:**
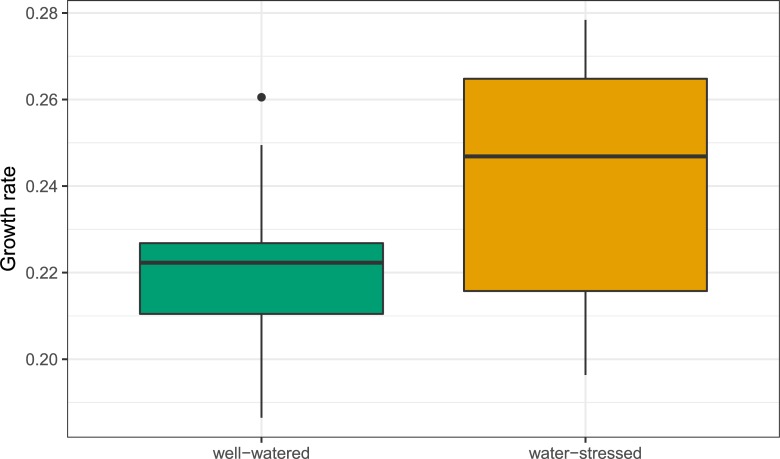
Larval growth rate (mg/d) of *Melitaea cinxia* on well-watered and water-stressed host plants. Larvae fed on well-watered and water-stressed host plant are displayed in green and orange fill, respectively.

Larvae had a markedly higher growth rate on water-stressed compared to well-watered host plants (*F*_1,29_ = 9.5, *P* = 0.004; Fig 1), while survival was unaffected by diet (*P*>0.3). The variance explained by the larval family was ~20% for growth rate and over 30% for survival (Table 1).

**Table 1:** Effect of plant water stress and bacterial infection on the larval life-history traits, immunity, and microbiota of *M*. *cinxia*. Significant *p*-values are given in bold.

Category	Response variable (statistical analysis method)	Family ICC^+^ and correlation	Fixed effect	Df.^ᶺ^	F / χ^2^ value	R^2^	*p-value*
Life-history traits	Growth rate (ANOVA)^1^	20.36	Drought	1,29 001	9 502	-	**0.0045**
	Survival (Chi-square)^1^^†^	33.79	Drought	1	0.794	-	0.373
Immunity	Active PO (ANOVA)^1^	0.00	Drought	1,36	0.495	-	0.486
			Infection	1,36	0.289	-	0.594
			Drought × Infection	1,36	0.370	-	0.547
	Lysozyme (ANOVA)^1^^†^	53.34	Drought	1,90.03	0.112	-	0.738
	Attacin (ANOVA)^1^^†^	56.00	Drought	1,89.08	0.196	-	0.659
	PGRP-LC (ANOVA)^1^^†^	36.78	Drought	1,94.08	1 981	-	0.163
	βGRP (ANOVA)^1^^†^	26.40	Drought	1,94.89	0.049	-	0.825
	proPO (ANOVA)^1^^†^	28.98	Drought	1,95.00	1 284	-	0.242
	pelle (ANOVA)^1^^†^	55.62	Drought	1,93.07	6 113	-	**0.015**
	serpin (ANOVA)^1^^†^	60.96	Drought	1,95.03	0.012	-	0.913
Microbiota	Fungal alpha diversity (ANOVA)^1^	52.90	Drought	1,27	2.563	-	0.121
			Infection	1,27	3.868	-	0.060
			Drought × Infection	1,27	1.744	-	0.198
	Bacterial alpha diversity (ANOVA)^1^	39.32	Drought	1,27	0.690	-	0.413
			Infection	1,27	1.117	-	0.300
			Drought × Infection	1,27	1.319	-	0.261
	Fungal community variations (*adonis-*ANOVA)^2^	25.70	Drought	1,36	1.425	0.037	**0.026**
			Infection	1,36	0.237	0.006	0.912
			Drought × Infection	1,36	0.658	0.017	0.406
	Bacterial community variations (*adonis*-ANOVA)^2^	8.37	Drought	1,36	0.669	0.018	0.333
			Infection	1,36	0.381	0.010	0.663
			Drought × Infection	1,36	0.910	0.948	0.181
	Fungal homogeneity of communities (HOMOVA)^2^	n.a.	Drought	1,38	0.014	-	0.918
			Infection	1,38	0.321	-	0.553
	Bacterial homogeneity of communities (HOMOVA)^2^	n.a.	Drought	1,38	10.071	-	**0.008**
			Infection	1,38	0.428	-	0.511

^1^ The family level was used as a random variable

^2^ The different family levels were used as strata to constrain permutations

^†^ Gene expression

^+^ Intraclass Correlation Coefficient

ᶺ Degrees of freedom

**Fig 2:**
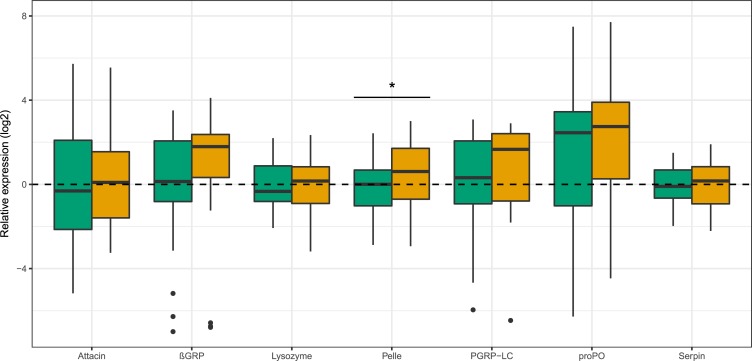
Immune gene expression of larvae fed on well-watered and water-stressed host plants. Larvae fed on well-watered and water-stressed host plants are displayed in green and orange fill, respectively.

PO activity was unaffected by larval diet or bacterial challenge (*P*<0.4 for both, Table 1). Pelle expression was upregulated by host plant water stress (*F*_1,93_ = 6.1, *P*<0.015; Table 1; Fig 2), while the remaining genes were unaffected (*P*>0.1 for all). Larval family considerably affected the variance in immune gene expression (Table 1).

**Fig 3:**
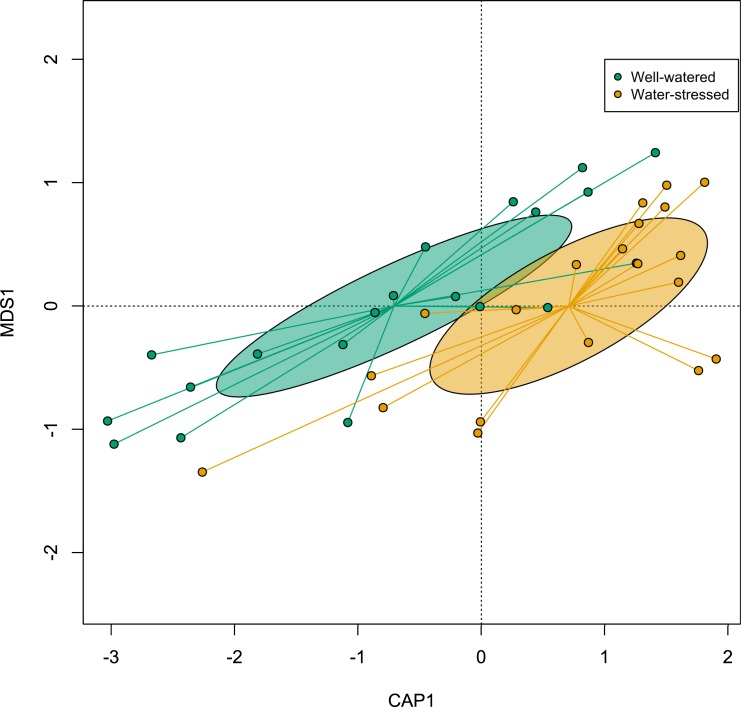
Canonical analysis of principal coordinates (CAP) of the fungal β-diversity among guts of *Melitaea cinxia* fed with well-watered or water-stressed host plants. Larvae fed on well-watered or water-stressed host plants are represented in green and orange respectively.

**Fig 4:**
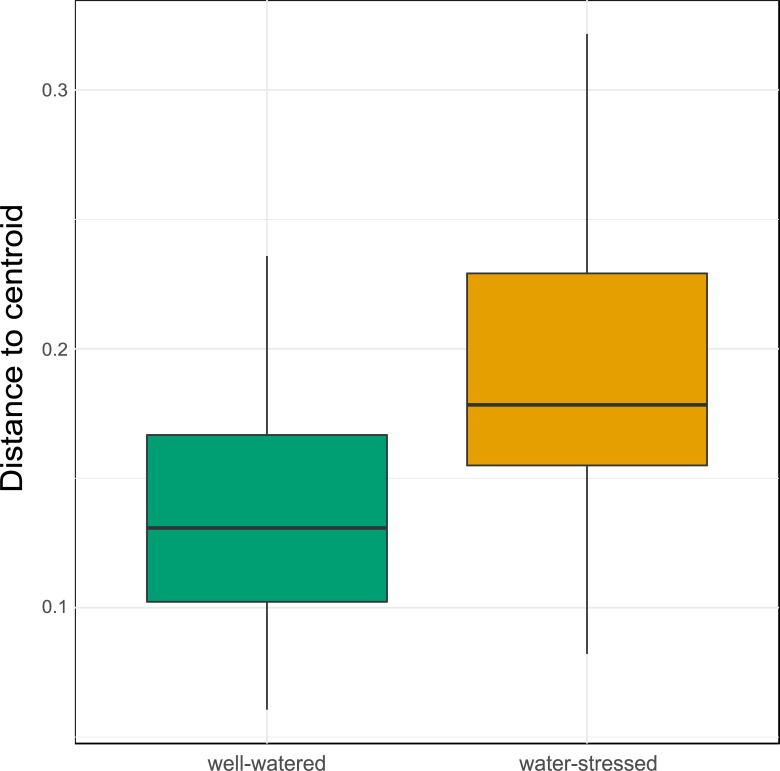
Heterogeneity of the bacterial community in the gut of *Melitaea cinxia*. Distances from the group centroid represent the heterogeneity of the bacterial community within each group. Larvae fed on well-watered or water-stressed host plants are represented in green and orange respectively.

The α-diversity of fungal and bacterial communities associated with *M*. *cinxia* did not respond to bacterial challenge or water stress treatment (Table 1). However, the fungal community was shifted (*adonis*-ANOVA; *F*_1,36_ = 1.425; *P* = 0.026; Fig 3) and the bacterial community was more heterogeneous (HOMOVA; *F*_1,38_ = 10.071; *P* = 0.008; Fig 4) in larvae fed with water-stressed plants, with no effect of bacterial challenge (Table 1). Larval family considerably impacted α-diversity (39.32% and 52.90% for bacterial and fungal communities, respectively), but only moderately affected its composition (8.37% and 25.70% for bacterial and fungal communities, respectively).

## Discussion

Despite the widespread concept of water stress negatively impacting insect population dynamics, there is growing evidence of more complex interactions between host plant condition and insect performance [2–4]. In line with the present day global warming trends, recent work has shown that population dynamics of the Glanville fritillary butterfly can be heavily impacted by drought or low precipitation events [24]. We found that larvae reared on mildly water-stressed host plants grew better with no impact on survival. One of the immune genes assessed, coding for pelle, was upregulated in larvae fed with water-stressed plants. The implications of this response are unclear, but the activation of an immune reaction toward infective microbes ingested with the food seems unlikely, given the absence of fitness costs on the water stress diet. Moreover, the microbial community of guts was more diverse in larvae fed on water-stressed plants. We are unaware of whether a more diverse gut microbial community is causally linked to a better larval growth, but we speculate that this possibility indeed exists: a previous study conducted on post-diapause instars showed that the microbiota composition explained about 50% of the variation in larval growth rate, suggesting that the two measures are correlated [20]. In general, our findings suggest a higher larval performance on moderately water-stressed plants. Intriguingly, larvae of the same age exposed to severe plant water stress have been reported to have a low performance (reduced survival and body mass) [36].

One well-documented effect of water stress on plants is an increase in the accumulation of amino acids and sugars in leaf tissues [37,38]. Potentially, the better growth rate of larvae found here could be explained in terms of increased nutrient accumulation or accessibility on water-stressed plants. However, this should be confirmed by future work formally quantifying macronutrient content in leaf tissues.

The immune gene coding for pelle was upregulated by larvae feeding on water-stressed host plants. Pelle is a protein of the Toll pathway, which is responsive to fungi, yeast and gram-positive bacteria [39]. Pelle is involved in the release of antimicrobial peptides (AMPs), defensive molecules which are only expressed upon infection [39]. We are unaware whether the activation of pelle eventually led to the production of antimicrobial peptides as the only candidate AMP gene tested here, attacin, has been shown to be most effective against gram-negative bacteria in Diptera [40] and in Lepidoptera [41], and hence not part of the Toll pathway featuring pelle. On the other hand, certain attacins can be active also against gram-positive bacteria or have no antimicrobial activity at all [42]. Indeed, the mechanism linking plant water stress and AMPs production remains to be explored, and its implications can only be speculated. On one hand, this may indicate that some potentially harmful microbes were detected in larvae fed on water-stressed plants, activating pelle and AMP production. The microbes causing this response may have been ingested with the food or their relative abundance in the gut may have been altered by the ingestion of water-stressed plant tissues. However, larvae fed on water-stressed plants had higher performance, which is unlikely in the presence of infectious microbes. On the other hand, larvae may have been only primed by microbes found on the water stress diet, but without the activation of an immune reaction featuring AMP production. In order to understand the significance of this response, future work involving detailed characterization of the microbial community taxa of both diets is needed.

Interestingly, larvae fed on water-stressed and well-watered plants had some differences in their gut microbiota composition: the bacterial community was more heterogeneous in the water stress treatment, while the fungal community was shifted, meaning that the fungal taxa composing the gut community differed almost completely between the two feeding treatments. In most Lepidoptera, the bacterial gut microbiota is mostly acquired through the food and is hence mostly transient [23]. Therefore, gut bacteria are suggested to have little impact on individual performance [23]. Yet, in few cases, bacterial symbionts have been shown to impact insect reproduction or their ability to be protected against pathogens [22]. Conversely, eukaryotic microorganisms interacting with Lepidoptera such as fungi are currently poorly characterized, and their impact on host performance is largely unknown. However, studies with other insect models suggest that fungi might play a role in nutrient-provisioning [43], steroid synthesis [44] or protection against pathogens [45]. Intestinal microorganisms are also known to modulate gut immunity of insect hosts [46]. In turn, insects control their microbiota composition by modulating specific immune pathways [46]. The effect of plant drought on fungal microbes found here suggests exciting perspective for future research on insect-plant interactions. With the present data, we are unable to say, however, whether the impact on microbiota composition and immune gene expression is driven directly by the food ingested, or indirectly via some microbiota-immunity interaction.

To conclude, we mostly found beneficial effects of moderate plant drought-stress on insect performance, indicating that the previously suggested negative impact of reduced precipitation is likely due to extreme desiccation [24]. This pattern is confirmed by studies finding reduced survival and body mass of larvae fed on severely water-stressed plants [36]. Our water stress treatment may, in fact, reflect a rather common condition in the field, where the butterfly often occupies open and dry outcrop meadows [47]. Notably, we found a more heterogeneous and diverse gut microbial community under the water stress treatment, and we speculate it potentially plaid a role in shaping the positive larval performance observed in terms of growth rate. More functional assays should be performed in the future to confirm or disprove this idea. Finally, the shift in the fungal community in response to diet detected here calls for future research on the understudied eukaryotic microbial community interacting with lepidopteran hosts.
